# Immune Signature-Based Risk Stratification and Prediction of Immunotherapy Efficacy for Bladder Urothelial Carcinoma

**DOI:** 10.3389/fmolb.2021.673918

**Published:** 2021-12-24

**Authors:** Fangfang Liang, Yansong Xu, Yi Chen, Huage Zhong, Zhen Wang, Tianwen Nong, Jincai Zhong

**Affiliations:** ^1^ Department of Medical Oncology, Guangxi Medical University First Affiliated Hospital, Nanning, China; ^2^ Emergency Department, Guangxi Medical University First Affiliated Hospital, Nanning, China; ^3^ College of Oncology, Guangxi Medical University, Nanning, China

**Keywords:** bladder urothelial carcinoma, immune-related genes, tumor immune microenvironment, immune checkpoint inhibitor, immunotherapy, prognostic model

## Abstract

Immune-related genes (IRGs) are closely related to tumor progression and the immune microenvironment. Few studies have investigated the effect of tumor immune microenvironment on the survival and response to immune checkpoint inhibitors of patients with bladder urothelial carcinoma (BLCA). We constructed two IRG-related prognostic signatures based on gene–immune interaction for predicting risk stratification and immunotherapeutic responses. We also verified their predictive ability on internal and overall data sets. Patients with BLCA were divided into high- and low-risk groups. The high-risk group had poor survival, enriched innate immune-related cell subtypes, low tumor mutation burden, and poor response to anti-PD-L1 therapy. Our prognostic signatures can be used as reliable prognostic biomarkers, which may be helpful to screen the people who will benefit from immunotherapy and guide the clinical decision-making of patients with BLCA.

## Introduction

Bladder cancer is the 10th most common cancer worldwide. In 2017, 474,000 new cases of bladder cancer were diagnosed and 197,000 deaths from the disease were reported worldwide (Fitzmaurice et al., 2019). Bladder urothelial carcinoma (BLCA) accounts for about 80–90% of all pathological types (Felsenstein and Theodorescu, 2018). About 25% of patients have muscle-invasive or metastatic lesions at the time of onset (Kamat et al., 2016). Patients have only 12–15 months of overall survival (OS) after being diagnosed before 2016 because of the lack of substantial progress in BLCA treatment; this finding has been maintained for the past 30 years (Bellmunt et al., 2017; Vlachostergios and Faltas, 2018). However, since 2016, several studies on immune checkpoint inhibitors (ICIs) have changed the treatment paradigm for metastatic urothelial cancer (mUC) and outlined a future therapeutic landscape (Rosenberg et al., 2016; Sharma et al., 2016; Apolo et al., 2017; Powles et al., 2017).

Considering that bladder cancer has high tumor mutation burden (TMB) and many mutations may be antigenic (Felsenstein and Theodorescu, 2018), traditional immunotherapy using *Bacillus* Calmette-Guérin has been successful for the treatment of early diagnosed bladder cancer (Kamat et al., 2015). Compared with previous first-line or second-line chemotherapy for mUC, new immunotherapy is undoubtedly an exciting breakthrough. However, the response rate of PD-1/PD-L1 inhibitors is only 20–24% (Kamat et al., 2015). PD-L1 status cannot highly predict treatment response; in this regard, more accurate predictive biomarkers should be identified to prescreen appropriate patients for immunotherapy and design personalized treatment. Existing immune and inflammatory markers in tumor may be the best biomarkers for evaluating the potential response of ICIs (Felsenstein and Theodorescu, 2018).

Studies have found that bladder cancer, similar to breast cancer, can be divided into different subtypes based on gene expression diversity and histological characteristics; this classification may be conducive to hierarchical management and precise treatment (Groenendijk et al., 2016; The Cancer Genome Atlas Research Network, 2014). Epigenetic regulatory mechanisms, such as DNA methylation, histone modification, and ncRNA expression, change with the development of bladder cancer and therefore may be used as potential biomarkers and therapeutic targets (Dudziec et al., 2011; Schulz and Goering, 2016; Peng et al., 2018). Additionally, differences in the molecular and genetic characteristics of tumor cells (such as gene mutation and copy number alteration) and the tumor microenvironment have a high impact on tumor invasiveness and sensitivity to treatment. The molecular profiles of immune components in the tumor microenvironment have great value as prognostic biomarkers. Immune-related genes (IRGs) can be quantified from a variety of cell types in a sample and have a predictive value for BLCA prognosis; these genes may be suitable biomarkers (Sweis et al., 2016; Li et al., 2020; Xu et al., 2020).

In this study, we downloaded the expression profiles and clinical information of patients from The Cancer Genome Atlas (TCGA)-BLCA cohort, analyzed the resulting differentially expressed genes (DEGs), and intersected the DEGs with the IRG sets downloaded from the ImmPort database (https://www.immport.org/home) to obtain differentially expressed immune-related genes (DE-IRGs). The DE-IRGs associated with prognosis were further analyzed to construct prognostic models. Furthermore, we assessed the differences in the immune cell infiltration, TMB, and response to ICI treatment between the high- and low-risk groups. The robust prognostic model may improve risk stratification and provide a more accurate assessment for the clinical management of BLCA.

## Materials and Methods

### Data Acquisition

The latest RNA-Seq expression profile data and clinical information of patients with BLCA were downloaded from TCGA (https://www.nature.com/articles/ng.2764, 2013), and data were also obtained from the Genotype-Tissue Expression (GTEx, https://www.gtexportal.org/home/) project. The lists of 456 IRGs and 318 transcription factors were derived from the ImmPort database (Sweis et al., 2016; Li et al., 2020; Xu et al., 2020) and Cistrome Cancer database (Mei et al., 2017), respectively, for subsequent analysis. The detailed clinicopathological and sequencing information of patients with advanced urothelial cancer treated with anti-PD-L1 agents (IMvigor210 cohort) were downloaded from the “IMvigor210CoreBiologies” R package (Mariathasan et al., 2018).

### DE-IRG Identification

The expression profiles from TCGA and GTEx portal were integrated using the “limma” R package (Ritchie et al., 2015) to calculate the DEGs between BLCA tumor tissues and normal tissues (FDR <0.05 and |log2FC| > 1). All DEGs and IRGS were crossed to obtain 172 DE-IRGs. GO and KEGG pathway enrichment analyses of the DE-IRGs were conducted using the “clusterProfiler” R package (v3.16.1) (Yu et al., 2012).

### Construction and Validation of OS and Disease-Free Survival Prognostic Signatures for Bladder Urothelial Carcinoma

Univariate Cox regression analysis was conducted to screen for DE-IRGs that are significantly associated with prognosis (*p <* 0.05). Least absolute shrinkage and selection operator (LASSO) Cox regression model (Tibshirani, 1996; Goeman, 2010) was used to construct an immune-related risk model using the “glmnet” R package as follows (Simon et al., 2011): Risk score = (level of gene a × coefficient a) + (level of gene b × coefficient b) + (level of gene c × coefficient c) + … + (level of gene *n* × coefficient *n*) (Yang et al., 2019). LASSO regression model is a compression estimation method, which constructs a penalty function to obtain a more refined model. This approach is a common method for biased data estimation with multicollinearity and has the advantage of preserving subset contraction. All samples were substituted into the formula to calculate the risk score value, which was then converted into Z-score using Z-score standardization. The samples with Z > 0 were included the high-risk group, and those with Z < 0 were included into the low-risk group. Kaplan–Meier survival curve analysis, log-rank test, and time-dependent Receiver operating characteristic (ROC) curve analysis were used to evaluate the predictive capability of the immune signatures.

### Functional Enrichment Analysis of Gene Set

The “limma” R package (v3.36.5) (Yang et al., 2019) was used to calculate the differences in gene expression between the high- and low-risk groups. Gene set enrichment analysis (GSEA) (Hänzelmann et al., 2013) was used to analyze the KEGG pathway enrichment for the gene set sorted by log2 fold change value.

### Estimation of the Tumor Microenvironment

CIBERSORT is a deconvolution analysis tool based on the principle of linear support vector regression for the expression matrix of human immune cell subtypes, which can infer the constituent ratio of 22 immune cell subtypes in complex tissues (Hänzelmann et al., 2013). The CIBERSORT computational tool (Chen et al., 2018) was used to determine the relative abundance of tumor-infiltrating immune cells.

### Evaluation of Tumor Mutation Load

The TCGA database was mined for TMB to analyze and visualize the mutation spectrum by “maftools” in R (Mayakonda et al., 2018). The mutation load score of each sample was calculated to compare the TMB difference between the two groups (calculation formula: $\text{TMB}=\;\frac{\text{total mutation}}{\text{total covered bases}}\;\times \;10^{6}$ ).

### Statistical Analysis

R software (v3.5.2) was used for statistical analysis. Differences between variables were analyzed by Chi-square test for categorical and continuous variables. Kaplan–Meier survival curve was constructed to compare survival across groups. ROC curve was used to determine the accuracy of the model. Statistical significance was set as *p* < 0.05.

## Results

### Clinical Information and Identification of Differentially Expressed Immune-Related Genes

After the RNA-seq data from TCGA-BLCA and GTEx was preprocessed, 407 tumor samples with OS information and 394 tumor samples with DFS information were obtained. The statistical results are shown in Supplementary Table S1. The patients with complete prognosis information were divided into training group (OS: *n* = 305, DFS: *n* = 295), testing group (OS: *n* = 102, DFS: *n* = 99), and entire group (OS: *n* = 407, DFS: *n* = 394) according to the ratio, 2:1:3. Chi-square test (*p* > 0.1) showed no difference in the distribution of clinical information between the testing and training groups (Table 1).

**TABLE 1:** The clinical characteristics and chi square test of each subgroup of BLCA patients.

		Patients with OS		X-squared	*p*-value	Patients with DFS		X-squared	*p*-value
		Training group	Testing group			Training group	Testing group		
Total		305	102			295	99		
OS/DFS	status_0	177	52	1.3	0.3	213	59	1.9	0.2
	status_1	128	50			82	40		
Age	Age > 60	223	77	0.12	0.7	216	75	0.13	0.7
	Age <= 60	82	25			79	24		
Gender	Female	75	31	1.1	0.3	72	29	0.69	0.4
	Male	230	71			223	70		
M_stage	M0	148	48	3.5	0.3	145	46	0.86	0.7
	M1	7	4			7	4		
	MX	148	49			141	48		
	unknown	1	2			2	1		
N_stage	N0	186	50	5.4	0.4	181	50	4.2	0.4
	N1	32	14			31	14		
	N2	52	23			49	21		
	N3	6	2			6	2		
	NX	24	12			23	11		
	unknown	5	1			5	1		
T_stage	T0	1	0	4.8	0.2	1	0	3.5	0.2
	T1	3	0			3	0		
	T2	94	15			84	25		
	T3	147	46			138	45		
	T4	36	22			36	20		
	TX	1	0			1	0		
	unknown	23	9			22	9		
Stage	stage_I	2	0	3.4	0.2	2	0	3	0.2
	stage_II	102	28			102	28		
	stage_III	107	32			101	31		
	stage_IV	93	41			89	39		
	stage_no	1	1			1	1		
smoke	smoke_yes	211	74	0.091	0.8	205	72	0.013	0.9
	smoke_NO	83	26			79	26		
	smoke_UN	0	13			11	1		

**FIGURE 1:**
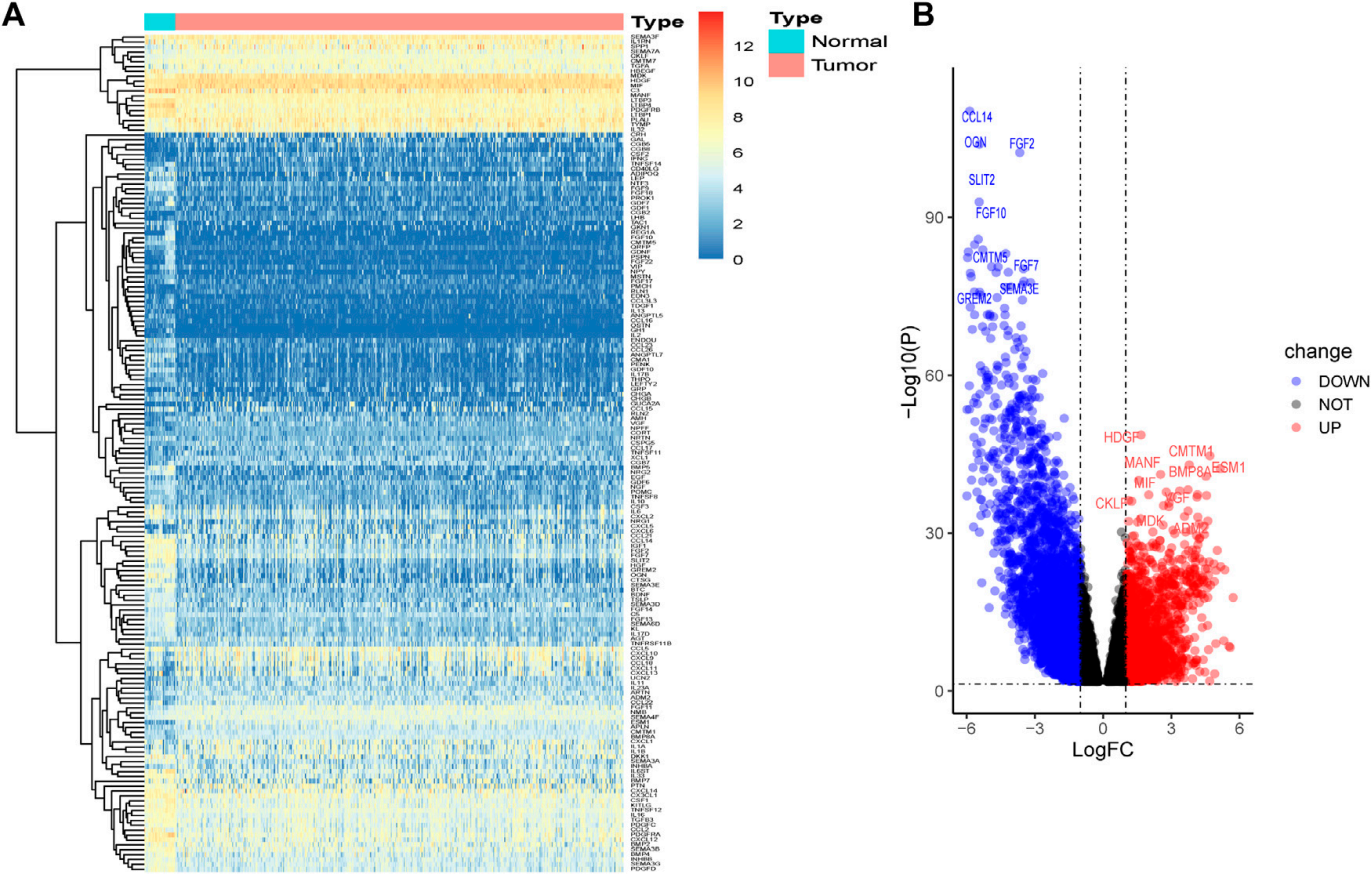
Identification of DE-IRGs between tumors and normal bladder tissues. **(A)** Heatmap of DE-IRGs. **(B)** Volcano plot of DE-IRGs.

A total of 1,854 upregulated genes and 3,145 downregulated genes were identified between tumor and normal tissues (Supplementary Figure S1). The intersection of the predicted DEGs with the IRGs in BLCA yielded 62 upregulated and 109 downregulated DE-IRGs (Figure 1).

### Biological Characteristics of Differentially Expressed Immune-Related Genes

**FIGURE 2:**
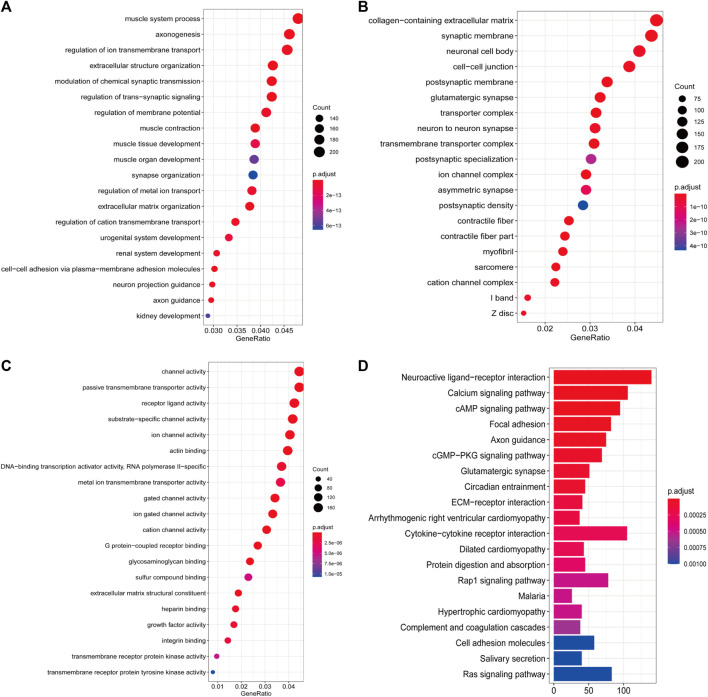
Enrichment analyses of DE-IRGs. **(A)** Top 20 most enriched BP categories. **(B)** Top 20 most enriched CC categories. **(C)** Top 20 most enriched MF categories. **(D)** Top 20 most enriched KEGG pathways.

Pathway enrichment analysis was performed to interpret the biological function of the DE-IRGs. A total of 195 GO categories were found with an FDR of less than 0.05. The most significant terms are displayed in Figures 2A–C. The GO analysis results showed that the enriched biological process (BP) terms were muscle system process and axonogenesis. The enriched cellular component (CC) terms were related to collagen-containing extracellular matrix and synaptic membrane. Among molecular function (MF) terms, channel activity and passive transmembrane transporter activity were dominant. For the KEGG pathway analysis (23 terms, FDR <0.05), the top 20 enriched terms included neuroactive ligand–receptor interaction, calcium signaling pathway, and cAMP signaling pathway (Figure 2D). We further studied the relationship between target gene regulation and transcription factors. First, 97 differentially expressed transcription factor-related genes (DE-TFs) were obtained from the intersection of all DE-IRGs and transcription factor-related (TF) genes in BLCA. Univariate Cox proportional hazard regression analysis was conducted using the Coxph function in the “survival” R package, and *p* < 0.05 was selected as the threshold to filter data. Thirty-one OS-related and 27 DFS-related DE-IRGs were screened, and the regulatory network between these genes and DE-TFs (Supplementary Figure S2) was constructed using Cytoscape (v3.7.1) software (Cline et al., 2007).

### Construction and Verification of Gene Immune Signatures in Bladder Urothelial Carcinoma

The training group was used to identify prognostic immune genes and construct prognostic risk models. The testing and entire groups were used to verify the predictive ability and robustness of the models. First, univariate Cox regression analysis was used to identify the candidate genes that are significantly associated with prognosis (*p* < 0.05). Sixteen genes were screened from 31 OS-related DE-IRGs, and 22 genes were screened from 27 DFS-related DE-IRGs. We then conducted LASSO Cox regression analysis to further compress the genes and found 13 OS-related IRGs and 15 DFS-related IRGs (Supplementary Figures S3, S4). Finally, multivariate Cox proportional hazard regression analysis was carried out, and two prediction models were established as follows:

For the 13 OS-IRGs: Risk score = (0.0252 × HGF) + (0.0014 × FGF9) + (0.1379 × INHBB) + (0.0791 × PLAU) + (0.0082 × IL17B) + (−0.0519 × CXCL5) + (0.0775 × SEMA4F) + (0.0446 × PDGFRB) + (0.0621 × LTBP1) + (−0.0545 × FGF18) + (−0.1726 × CCL17) + (0.0618 × CGB8) + (0.1069 × CCL26) + (0.0550 × EGF) + (−0.0196 × CXCL1).

For the 15 DFS-IRGs: Risk score = (0.0252 × HGF) + (0.0014 × FGF9) + (0.1379 × INHBB) + (0.0791 × PLAU) + (0.0082 × IL17B) + (−0.0519 × CXCL5) + (0.0775 × SEMA4F) + (0.0446 × PDGFRB) + (0.0621 × LTBP1) + (−0.0545 × FGF18) + (−0.1726 × CCL17) + (0.0618 × CGB8) + (0.1069 × CCL26) + (0.0550 × EGF) + (−0.0196 × CXCL1).

**FIGURE 3:**
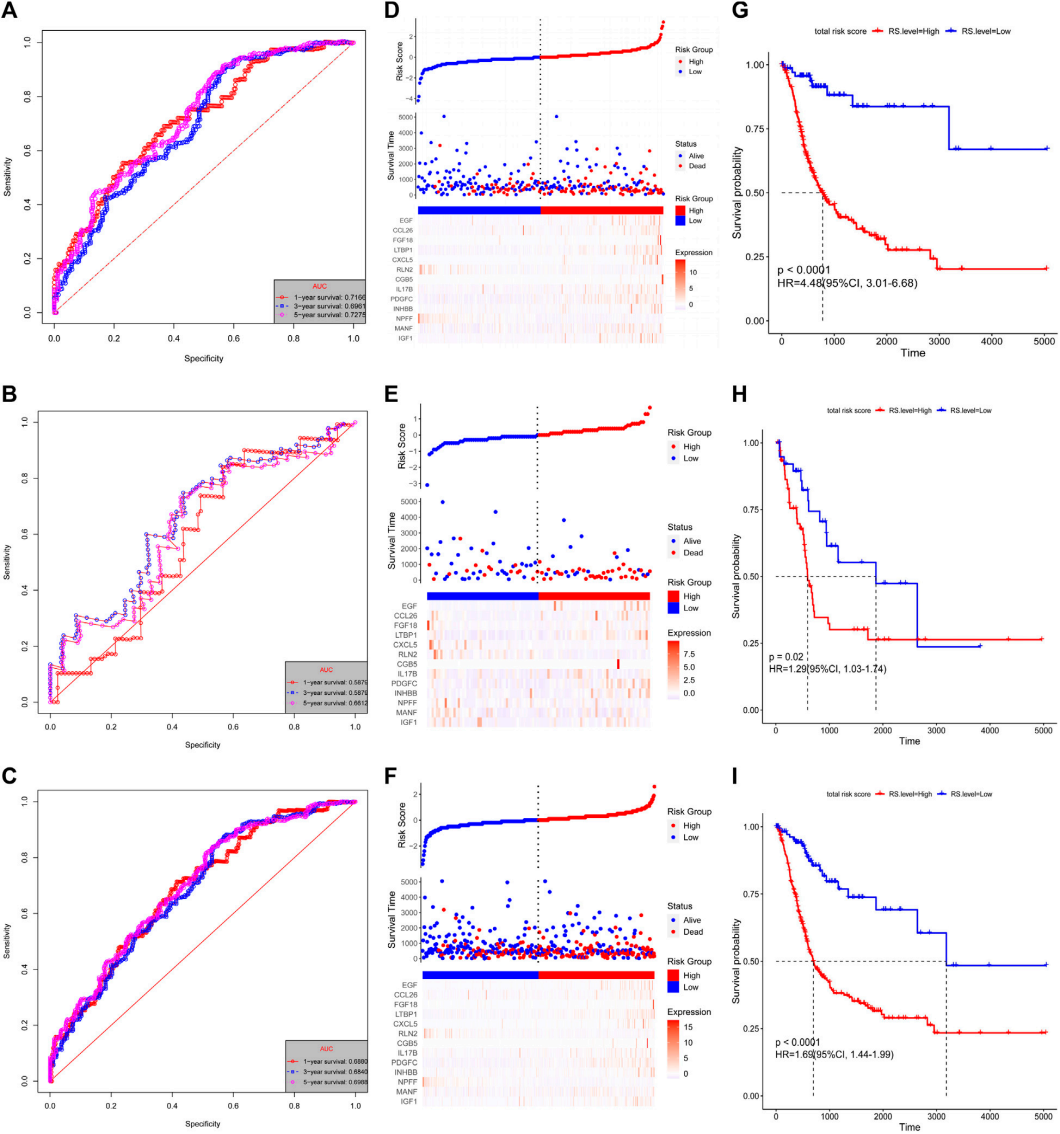
Validation of the 13-IRG signature. Kaplan–Meier estimates of the OS of the training group **(A)**, testing group **(B)**, and entire group **(C)**. Risk score distributions and heatmaps of the mRNA expression of the 13-IRG signature in the training group **(D)**, testing group **(E)**, and entire group **(F)**. Time-dependent ROC analyses of the 13-IRG signature in the training group **(G)**, testing group **(H)**, and entire group **(I)**.

**FIGURE 4:**
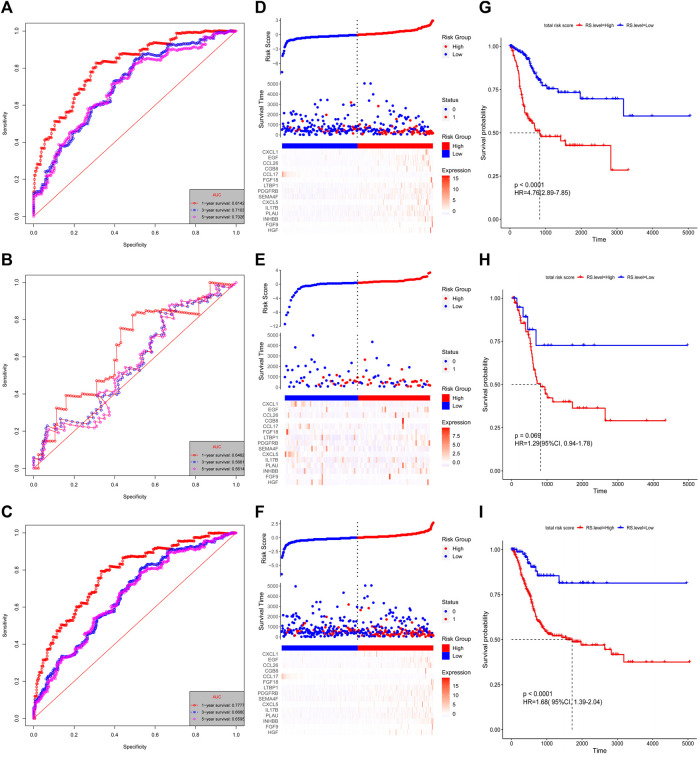
Validation of the 15-IRG signature. Kaplan–Meier estimates of the DFS of the training group **(A)**, testing group **(B)**, and entire group **(C)**. Risk score distributions and heatmaps of the mRNA expression of the 15-IRG signature in the training group **(D)**, testing group **(E)**, and entire group **(F)**. Time-dependent ROC analysis of the 15-IRG signature in the training group **(G)**, testing group **(H)**, and entire group **(I)**.

The risk score for each patient was calculated using these formulas. The patients with complete OS/DFS information were divided into the high-risk groups (OS: *n* = 294, DFS: *n* = 308) and low-risk groups (OS: *n* = 113, DFS: *n* = 86). The ROC analysis results revealed that the three groups had large areas under the curve (AUCs) for 1-, 3-, and 5-years survival (Figures 3A–C and Figures 4A–C). The risk score distribution maps (Figures 3D–F and Figures 4D–F) show a remarkable increase in the number of deaths in the high-risk group. This finding indicates that the samples with high-risk scores have a poor OS. In the training group, the Kaplan–Meier curves for OS and DFS consistently showed that patients in the low-risk group exhibited a better prognosis than patients in the high-risk group (Figure 3G and Figure 4G). The results were verified in the testing and entire groups (Figures 3H,I and Figures 4H,I). The results of the analyses of the three groups were consistent, which suggests the good predictive ability and robustness of the two models. In addition, the 13-IRG signature identified *EGF*, *CCL26*, *FGF18*, *LTBP1*, *CXCL5*, *CGB5*, *IL17B*, *PDGFC*, *INHBB*, *MANF*, and *IGF1* as risk factors, and their high expression was associated with high risk. By contrast, *RLN2* and *NPFF* were protective factors, and their high expression was associated with low risk. For the 15-IRG signature, 14 genes were protective factors (*HGF*, *FGF9*, *INHBB*, *PLAU*, *IL17B*, *CXCL5*, *SEMA4F*, *PDGFRB*, *LTBP1*, *FGF18*, *CGB8*, *CCL26*, *EGF*, and *CXCL1*), and one gene was identified to be a risk factor (*CCL17*).

### Potential Biological Functions of Different Genes in High- and Low-Risk Groups

The KEGG pathway-based GSEA of the DEGs between the high- and low-risk groups revealed potential biological importance (the top 10 pathways are shown in Supplementary Figure S5). In OS and DFS models, “cell adhesion molecules cams,” “chemokine signaling pathway,” “cytokine–cytokine receptor interaction,” and “JAK–STAT signaling pathway” were enriched in both gene sets. Hence, the key DEGs may be involved in tumor metastasis and immunosuppression.

### Prognostic Value of Immune Gene Signatures in Patients With Bladder Urothelial Carcinoma

Univariate and multivariate Cox regression analyses were conducted to systematically analyze the clinical information (including age, gender, T stage, N stage, M Stage, and smoking level) and risk score (high vs. low) of patients with BLCA in the training and entire groups (Table 2 and Supplementary Table S2). The univariate Cox regression analysis showed that a high-risk score was unfavorable for OS and DFS. The multivariate Cox regression analysis showed that the risk scores of the immune signatures were a remarkable predictor of survival. Therefore, our 13- and 15-IRG signatures had good predictive performance in the clinic.

**TABLE 2:** Univariate and multivariate Cox analysis in each group of BLCA patients with OS.

Variables	Univariate analysis		*p*-value	Multivariate analysis		*p*-value
	HR	95% CI		HR	95% CI	
training group						
Age (<60 vs. ≥ 60)	1.039	1.020–1.059	0	1.035	1.015–1.055	0
Gender (female vs. male)	0.84	0.571–1.236	0.382	0.889	0.600–1.316	0.557
Tstage (T1/T2 vs. T3/T4)	1.381	1.013–1.882	0.034	0.743	0.515–1.073	0.113
Nstage (N0 vs. N1/N2/N3)	1.378	1.155–1.644	0.001	0.952	0.705–1.286	0.747
Stage (I/II vs. III/IV)	1.693	1.350–2.123	0	1.688	1.117–2.553	0.013
Smoke_level (YES vs. NO)	1.071	0.778–1.473	0.672	1.023	0.741–1.411	0.892
Riskscore (high/low)	4.82	3.246–7.159	0	3.804	2.528–5.724	0
entire group						
Age (<60 vs. ≥ 60)	1.033	1.017–1.049	0	1.031	1.015–1.047	0
Gender (female vs. male)	0.891	0.642–1.236	0.492	1.01	0.713–1.430	0.957
Tstage (T1/T2 vs. T3/T4)	1.487	1.140–1.941	0.002	0.889	0.655–1.207	0.451
Nstage (N0 vs. N1/N2/N3)	1.385	1.196–1.605	0	0.951	0.741–1.221	0.695
Stage (I/II vs. III/IV)	1.683	1.393–2.034	0	1.668	1.193–2.332	0.003
Smoke_level (YES vs. NO)	1.219	0.909–1.634	0.176	1.144	0.849–1.540	0.377
Riskscore (high/low)	1.693	1.438–1.993	0	1.576	1.303–1.906	0

**FIGURE 5:**
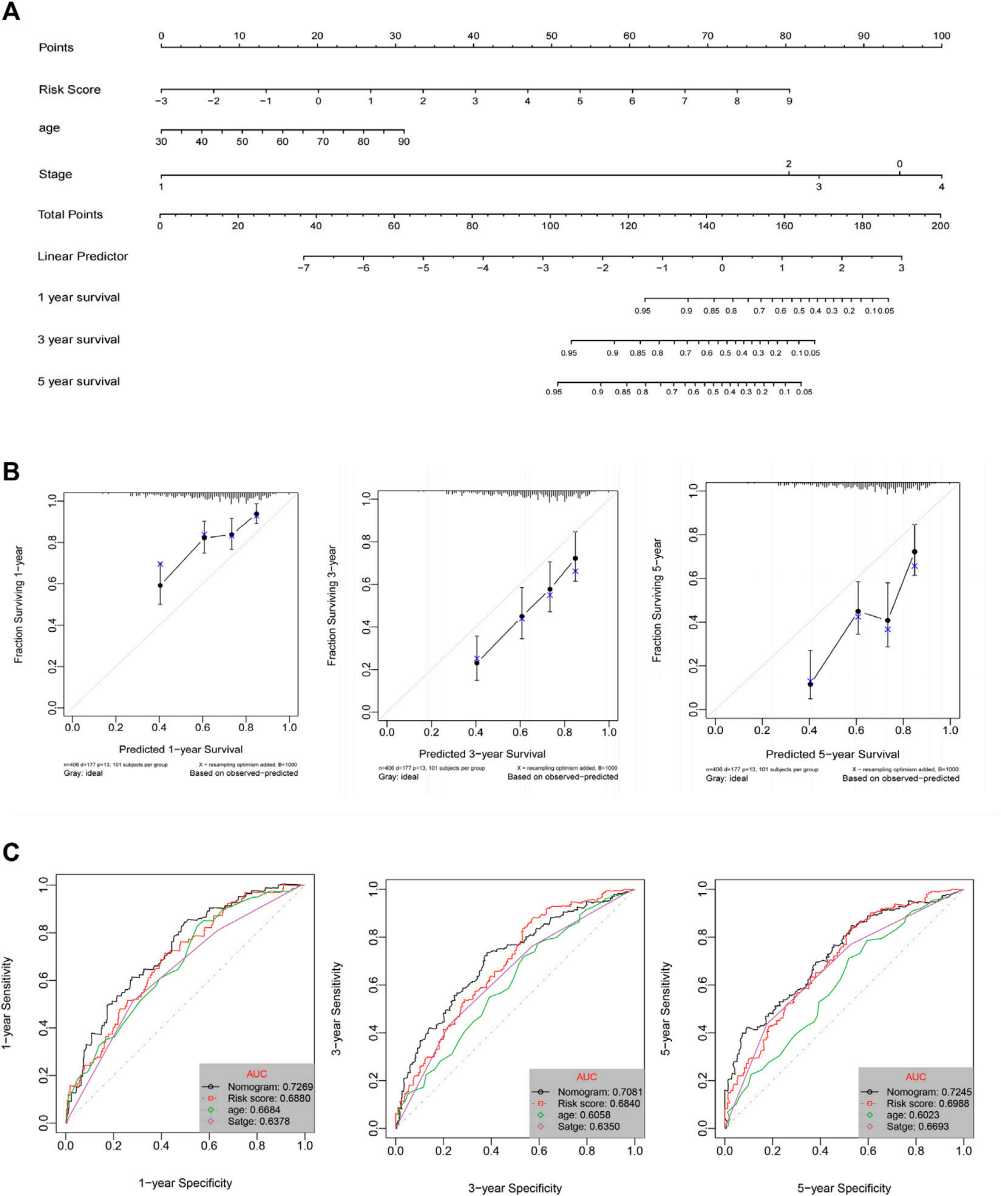
**(A)** Nomogram prediction of OS probability. **(B)** Calibration plots of the nomogram for 1-, 3-, and 5-years OS prediction. **(C)** Time-dependent ROC curves of 1-, 3-, and 5-years OS.

A prognostic nomogram for OS/DFS with scales for three independent prognostic factors, including age, stage, and risk score, was constructed according to the results of the multivariate analysis. The nomogram displayed that risk score had the greatest impact on the prediction of survival rate (Figure 5A and Supplementary Figure S6A). This finding further confirmed that the immune signatures could predict prognosis. Calibration plots were used to visualize the predictive performance of the nomograms. Figure 5B shows that the calibration plots of 1-, 3-, and 5-years OS nomograms exhibited good performance and accurately estimated mortality. The DFS results are shown in Supplementary Figure S6B. The time-dependent ROC curves indicated the accuracy of the nomograms. The AUCs of 1-, 3-, and 5-years OS were 0.70, 0.653, and 0.723, respectively (Figure 5C), and the 1-, 3-, and 5-years DFS were 0.698, 0.751, and 0.70, respectively (Supplementary Figure S6C). In conclusion, risk score had better predictive ability compared with a single clinical factor. Moreover, the combined model of risk score and clinical factor showed the highest predictive accuracy.

**FIGURE 6:**
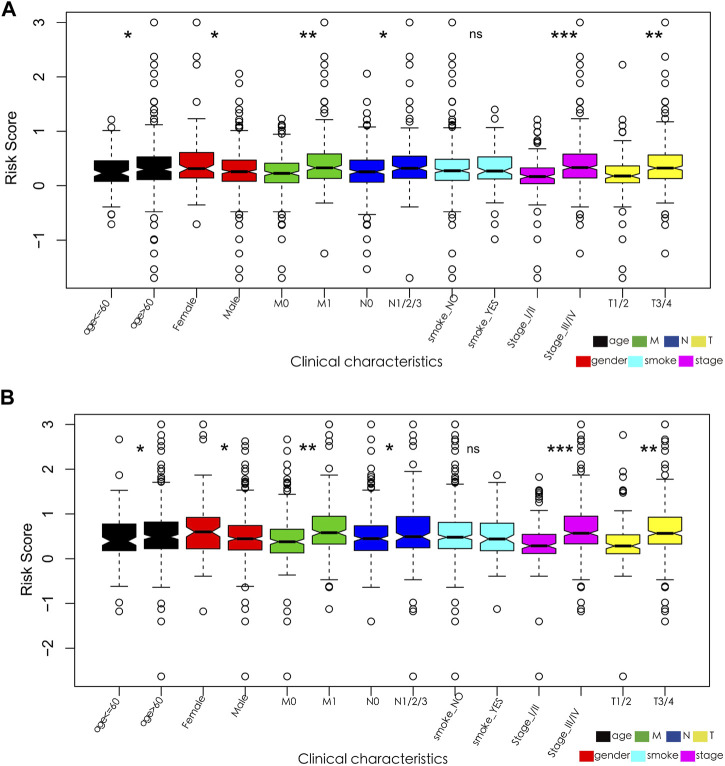
**(A)** Relationships between the 13-IRG signature and clinicopathological parameters. **(B)** Relationships between the 15-IRG signature and clinicopathological parameters.

In addition, the relationship between the characteristics of the 13- and 15-IRG signatures and clinicopathological parameters was analyzed. The results suggest that the risk score of patients >60 years, female, with lymph node metastasis and/or distant metastasis, T3/T4, and stage III/IV is substantially increased (Figure 6).

### Immune Gene Signatures and Tumor Immune Microenvironment

**FIGURE 7:**
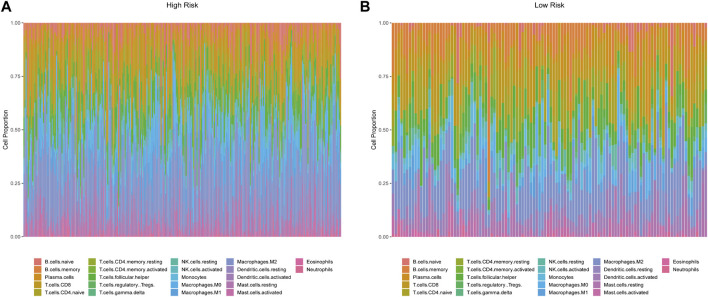
Characterization of immune cell infiltrate in high-risk **(A)** and low-risk groups **(B)** based on the 13-IRG signature.

Our study revealed the possible interaction and correlation between these identified IRGs and the tumor immune microenvironment. Differences in immune cell infiltration were found between the high- and low-risk groups of the OS model (Figure 7). In the high-risk group, the proportions of naïve B cells, CD4 memory T cells, macrophage M0, macrophage M1, and neutrophils were considerably higher, whereas the abundance of memory B cells and T naïve CD4 cells increased substantially in the low-risk group. In the DFS model, CD4 memory T cells, macrophage M1, neutrophils, and memory B cells showed the same distribution characteristics (Supplementary Figure S7). Multiple innate immune-related cell types, including macrophage M0, macrophage M1, and neutrophils, were enriched in the high-risk group, which may indicate adverse clinical outcomes.

### Immune Gene Signatures and Tumor Mutation Burden

**FIGURE 8:**
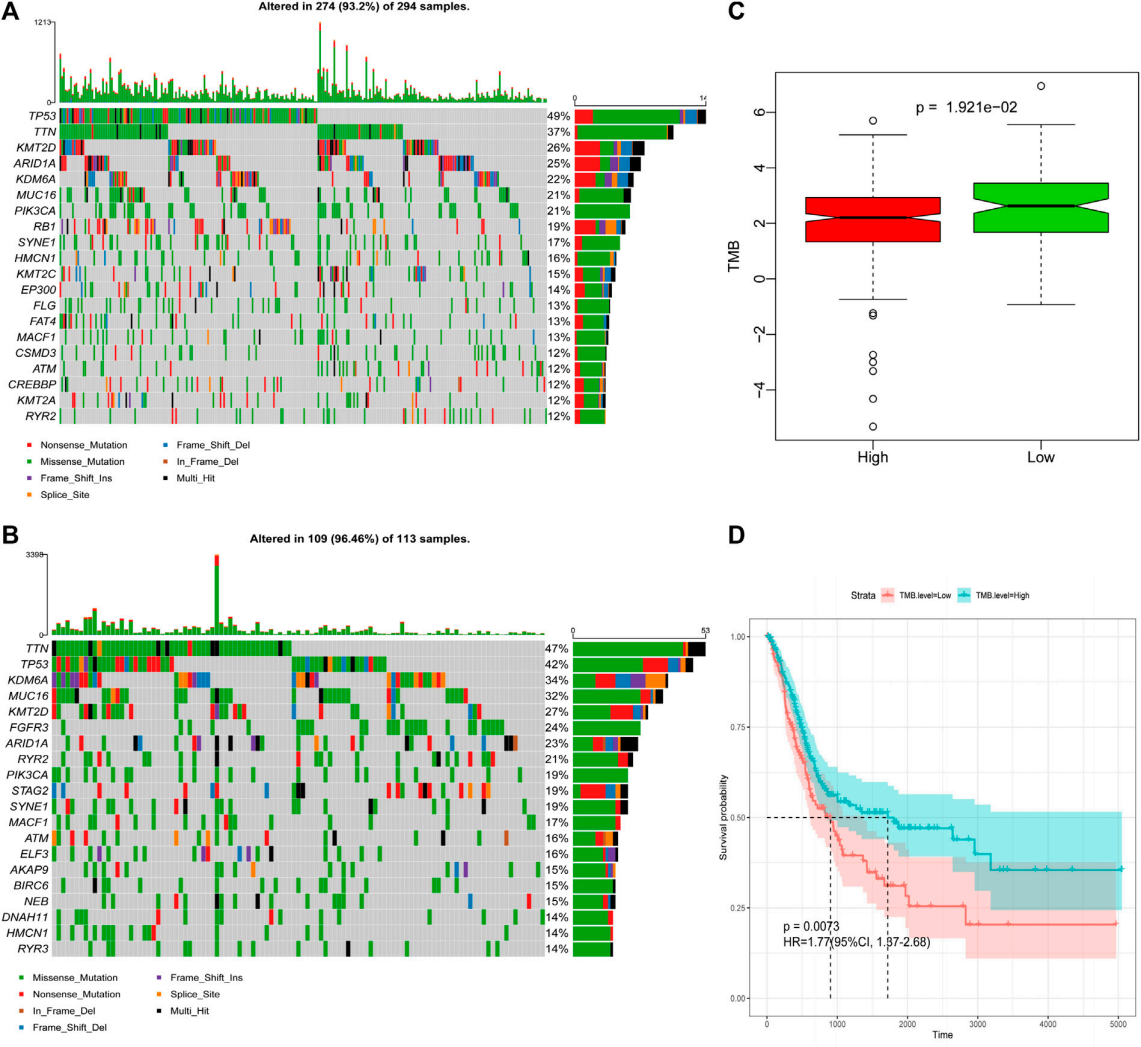
Differences in TMB between the high-risk and low-risk groups based on OS. **(A)** Mutation profile of high-risk group. **(B)** Mutation profile of low-risk group. **(C)** Box plot of TMB score in high-risk and low-risk groups. **(D)** Survival curves of high- and low-TMB score groups.

The frequency of mutation was high in all samples (>90%), and the main type of mutation was missense mutation. The mutation frequency of tumor suppressor gene, *TP53* (tumor protein p53), was the highest in the high-risk group, whereas the mutation frequency of titin was the highest in the low-risk group (Figures 8A,B, Supplementary Figures S8A,B). The box plot of TMB scores shows that the low-risk group had higher TMB and longer OS than the high-risk group (Figure 8C), and the corresponding DFS model analysis did not indicate the same results (Supplementary Figure S8C). However, after stratification according to the TMB of the sample, the Kaplan–Meier survival curve demonstrated that the difference between the two groups was statistically significant (Figure 8D, Supplementary Figure S8D).

### Prediction of Anti-PD-L1 Response With the Immune Gene Signatures

**FIGURE 9:**
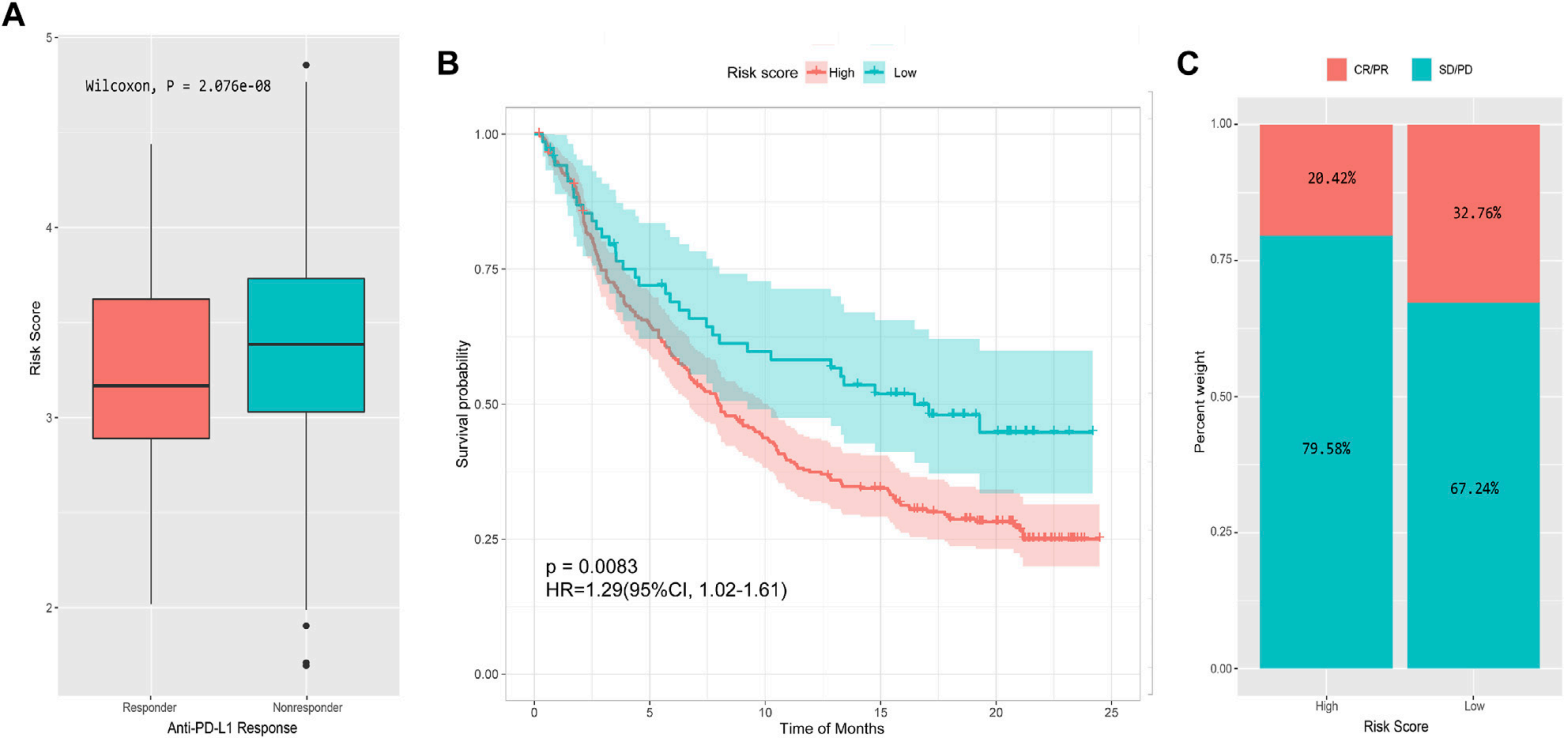
Performance of the 13-IRG signature in predicting response to anti-PD-L1 therapy in the IMvigor210 cohort. **(A)** Box plot of risk score and objective response to immunotherapy. **(B)** Survival curve of high- and low-risk groups. **(C)** Immunotherapy response rates of high- and low-risk groups.

The analysis of the real ICI treatment cohort confirmed the predictive value of the immune signatures for checkpoint immunotherapy. We downloaded the gene expression profiles and clinical data of the IMvigor210 cohort. The IMvigor210 study is a single arm, multicenter, phase 2 clinical trial that investigated the clinical activity of PD-L1 blockade with atezolizumab in mUC. A total of 298 pre-treatment tumor samples were used for transcriptome RNA sequencing to evaluate the integrated biomarkers (Cline et al., 2007). Considering the absence of DFS data, we only explored the use of OS-related risk signature in predicting the benefit of anti-PD-L1 therapy for urothelial carcinoma. All samples were divided into high- and low-risk groups by the OS risk model. The risk score of patients with treatment response [complete response (CR) or partial response (PR)] was significantly lower than that of patients without treatment response [stable disease (SD) or progressive disease (PD); Wilcoxon, *p* = 2.076e-08; Figure 9A]. Moreover, the prognosis of the low-risk group was significantly better than that of the high-risk group (*p* = 0.0083, Figure 9B). After evaluating the distribution of CR/PR and SD/PD in the high- and low-risk groups, we found that patients with low-risk scores had better response to ICI treatment than patients with high-risk score (Figure 9C).

## Discussion

The main aim in this study was to construct a model using IRGs to predict the prognosis of patients BLCA, as well as the clinical benefit of immunotherapy. In addition, we performed GO and KEGG pathway enrichment analyses for the DE-IRGs and further screened the DE-IRGs associated with OS or DFS to construct network interaction relationships with DE-TFs to explore the possible biological mechanisms of DE-IRGs associated with prognosis. We constructed 13- and 15-IRG signatures using the RNA-Seq data of TCGA-BLCA and the immune-related gene set from the ImmPort database, respectively. The univariate Cox regression analysis showed that the risk scores of the immune signatures were an independent predictor of survival. We next constructed a prognostic nomogram of OS/DFS using three variables (age, stage, and risk score), which showed good discrimination and prediction ability. Besides, we also found that the low-risk score group had better response to ICI therapy than the high-risk score group, and the two groups showed differences in immune cell infiltration and TMB.

Previous studies indicated that IRGs are involved in shaping the immune landscape and influence the prognosis and response to immunotherapies of patients with tumor (Liu et al., 2020; Yi et al., 2021). In the present study, the IRGs identified were related to the progression of malignant tumors, and some of them can regulate the occurrence and development of cancer by simultaneously regulating the state of the tumor immune microenvironment and the malignant biological characteristics of tumor cells. Yeh et al. (2015) found that fibroblast ERα increased the expression of CCL cytokines and IL-6 in the tumor microenvironment and promoted bladder cancer invasion. CXCL5 upregulates the expression of MMP2/MMP9 by activating PI3K/AKT signal to promote the migration and invasion of bladder cancer cells (Gao et al., 2015). In addition, CXCL5 is involved in changing the tumor microenvironment of bladder cancer. The interaction between endothelial cells and bladder cancer cells potentiates the recruitment of vascular endothelial cells through the CXCL1/CXCL5/CXCL8–CXCR2 pathway, which leads to tumor progression (Gao et al., 2015). Furthermore, research had found that *Salmonella* local immune stimulation considerably enhanced the expression of complement component 5a, CXCL2, CXCL5, CCL5, and CCL8; thus, it recruits specific CD8 T cells and promotes bladder cancer progression (Domingos-Pereira et al., 2015). Similarly, based on the principle of gene–immune interaction, CXCL5 plays an important role in the progression of colon cancer, gastric cancer, liver cancer, and other tumors by recruiting or activating neutrophils. A large number of studies have reported that the abnormal expression of epidermal growth factor (EGF) and its receptor is involved in the invasion and metastasis of a variety of tumors (Ma et al., 2012; Perera and Bardeesy, 2012; Tomas et al., 2014; Chen et al., 2021), such as bladder cancer, gastric cancer, liver cancer, breast cancer, and melanoma. Heparin-binding EGF-like growth factor (HB-EGF) accumulates in the nucleus of invasive bladder transitional cell carcinoma, which can promote the autocrine cycle of cells, lead to the proliferation of cancer cells, and protect cancer cells from apoptosis (Kim et al., 2005). Moreover, as a powerful tumor growth and angiogenesis inducer, HB-EGF promotes the migration of bladder cancer cells by inducing MMP-9 and MMP-3 expression and activity (Ongusaha et al., 2004). Therefore, a prognosis model constructed using these genes would have relatively sufficient basis, and our follow-up study confirmed this hypothesis.

Considering that studying the biological mechanism and function of gene sets involved in specific pathways is an effective method for cancer research (Ge et al., 2018; Liu et al., 2018), we conducted a functional enrichment analysis of the identified DE-IRGs in BLCA tumor tissues. The KEGG analysis indicated that DEGs in the high- and low-risk groups were enriched in immune-related pathways, such as “cell adhesion molecules,” “chemokine signaling pathway,” “cytokine–cytokine receptor interaction,” “JAK–STAT signaling pathway,” “leukocyte transendothelial migration,” and “natural killer cell-mediated cytotoxicity.” The results may indicate that related pathways are involved in the shaping of the immune landscape. These results provide new insights into the potential biological mechanism and function of IRGs.

Based on the principle of gene–immune interaction, we designed OS and DFS immune signatures to predict the prognosis and response to ICI of patients with BLCA. According to the univariate and multivariate Cox regression analyses, the risk scores of the immune signatures were an independent prognostic indicator of OS/DFS for patients with BLCA; that is, patients with low immune signature scores have a better prognosis. The nomogram and ROC analyses further verified the prediction performance of the IRG signatures. Risk score was also remarkably correlated with sex, age, and TNM stage; that is, patients with worse clinicopathological characteristics had higher risk scores. Through comprehensive analysis, we demonstrated that the IRG signatures might be a suitable guide for clinicians in conducting the risk stratification of patients with BLCA and could help to adopt appropriate treatment modes.

Tumor cells escape from immune surveillance by suppressing the effect of T cells through the immune checkpoint, which leads to a decrease in tumor surveillance and tumor recognition and the occurrence of immune escape (Gervois et al., 1996). Two key immune checkpoint receptors, namely, cytotoxic T lymphocyte antigen 4 (CTLA-4) and PD-1, have been widely used in emerging immunotherapy. The accurate mode of action and the determination of predictive markers are key research topics because of the minority of patients who appear to benefit from immunotherapy. Several biomarkers have been developed at the genome, transcriptome, and immunogenome levels (Havel et al., 2019). The expression of PD-L1 in tumor cells is a biomarker for predicting response to anti-PD-1 or anti-PD-L1 therapy, but data remain insufficient. Patients who tested negative for PD-L1 can still achieve an objective remission rate of 11–20% (Mahoney and Atkins, 2014; Rui et al., 2019). Tumor-infiltrating lymphocytes (TILs) (Mahoney and Atkins, 2014; Rui et al., 2019), TMB (Yarchoan et al., 2017; Wang et al., 2020), and microsatellite instability (Pećina-Šlaus et al., 2020) are related to therapeutic response to ICI treatment. Our study found that the IRG signatures were associated with immune cell infiltration and TMB, and their predictive effects were verified in the immunotherapy cohort.

The enrichment of TIL subsets associated with adaptive immunity decreased with tumor progression, whereas that associated with innate immunity increased (Charoentong et al., 2017). Using our analytical strategy, we found a similar evolving nature in infiltrating immune cell components during tumor progression; that is, TIL subpopulations related to innate immunity, such as macrophage M0, macrophage M1, neutrophils, and resting dendritic cells, were enriched in the high-risk group, whereas TILs related to adaptive immunity, such as memory B cells, naive CD4 T cells, and gamma delta T cells (Tγδ), were enriched in the low-risk group. Therefore, we speculate that the 13- and 15-IRG sets for model construction may change the biological behavior and therapeutic response of tumor cells by changing the tumor microenvironment. The predictive role of TMB in ICI treatment has been confirmed in many clinical studies (Davoli et al., 2017). Tumor cells with high TMB can produce more tumor-specific antigens and thus can be easily recognized and killed by immune cells (Kakoti et al., 2020). In our OS model, the TMB of the low-risk group was higher and the prognosis of the high-TMB group was remarkably better after the samples were grouped according to TMB. This finding suggests that a low-risk score predicts a good response rate to PD-1 inhibition and satisfactory clinical outcomes.

The IMvigor210 cohort study confirmed the above results. Compared with the PD-1 treatment nonresponse group, the risk score of the response group was considerably lower, and the low-risk group had a higher response to anti-PD-1 treatment and better prognosis. These data further supported that the IRG signatures may serve as a biomarker to predict the prognosis of patients with BLCA and their response to immunotherapy.

This study has some limitations. First, the 13- and 15-IRG signatures only used a series of immune genes, which are nonspecific to the specific immune microenvironment of patients with urothelial carcinoma. Second, the factors influencing tumor progression and immunotherapy are very complex, and the influence of immune genes may only be a part of them. Third, basic experiments and studies with larger sample sizes are needed to verify these associations. Despite these limitations, our analysis showed that the 13- and 15-IRG signatures can effectively predict the prognosis and response to ICI of patients with BLCA.

In summary, the 13- and 15-IRG signatures may be helpful to determine the prognosis of patients with BLCA and stratify those who will benefit from checkpoint blockade immunotherapy. This finding may contribute to cancer immunotherapy and promote the development of precise immune oncology.

## Data Availability

The datasets presented in this study can be found in online repositories. The names of the repository/repositories and accession number(s) can be found in the article/Supplementary Material.

## Abbreviations

AUCs, areas under the curve; BLCA, bladder urothelial carcinoma; BP, biological process; CR, complete response; CTLA-4, cytotoxic T lymphocyte antigen 4; CC, cellular component; DEGs, differentially expressed genes; DE-IRGs, differentially expressed immune-related genes; DFS, disease-free survival; DE-TFs, differentially expressed transcription factor-related genes; EGF, epidermal growth factor; GSEA, gene set enrichment analysis; GTEx, Genotype-Tissue Expression; HB-EGF, Heparin-binding EGF; IRGs, immune-related genes; ICIs, immune checkpoint inhibitors; LASSO, least absolute shrinkage and selection operator; MF, molecular function; mUC, metastatic urothelial cancer; PR, partial response; PD, progressive disease; ROC, receiver operating characteristic; SD, stable disease; TMB, tumor mutation burden; TCGA, The Cancer Genome Atlas; TF, transcription factor-related; TILs, Tumor-infiltrating lymphocytes.
