# Supplementary figures and images for: Immune Signature-Based Risk Stratification and Prediction of Immunotherapy Efficacy for Bladder Urothelial Carcinoma
Source: Front Mol Biosci. 2021 Dec 24;8:673918. doi: 10.3389/fmolb.2021.673918 (PMC8739239; doi:10.3389/fmolb.2021.673918)

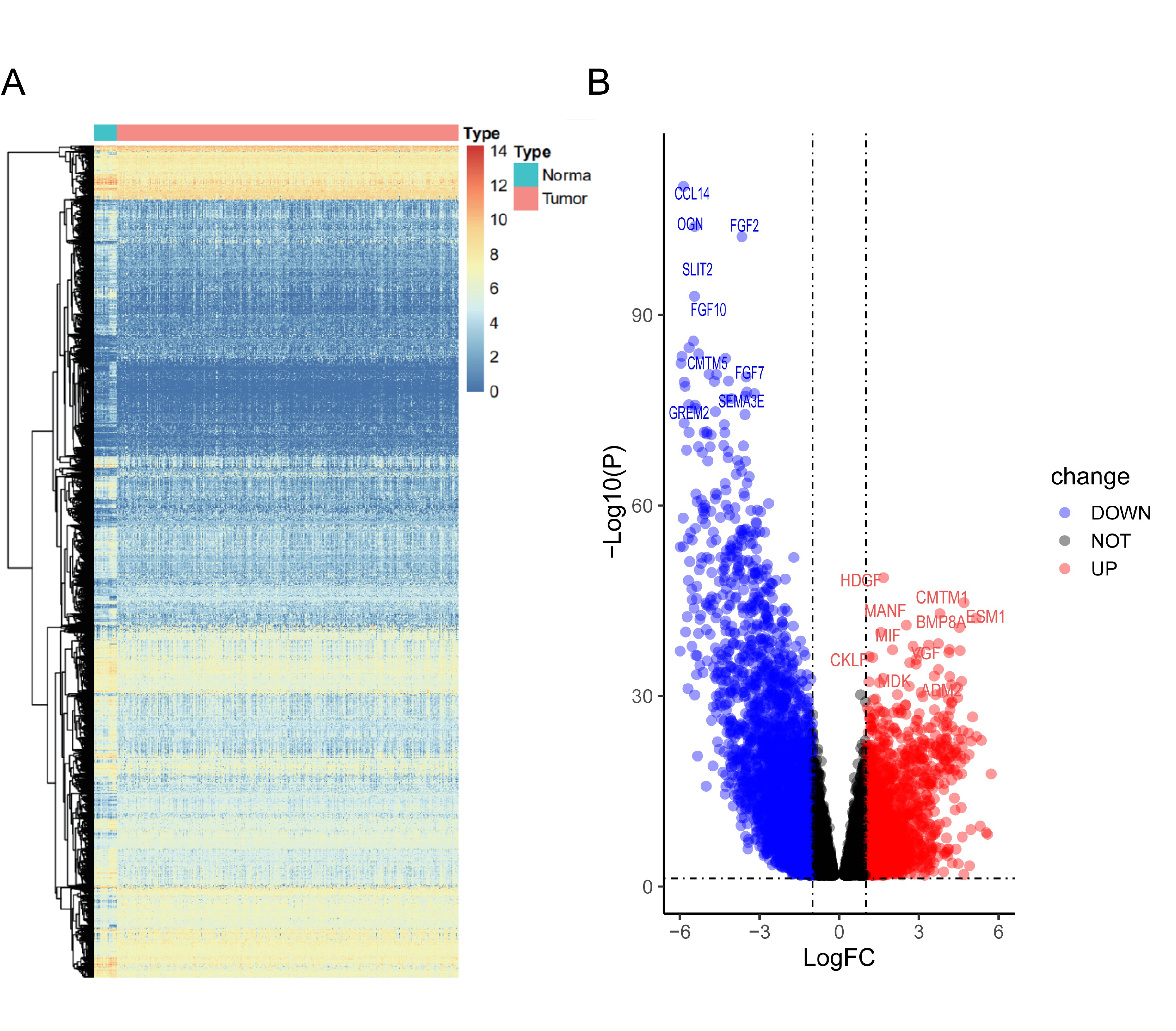

Supplement: Supplementary file 1 [file Image1.TIFF]

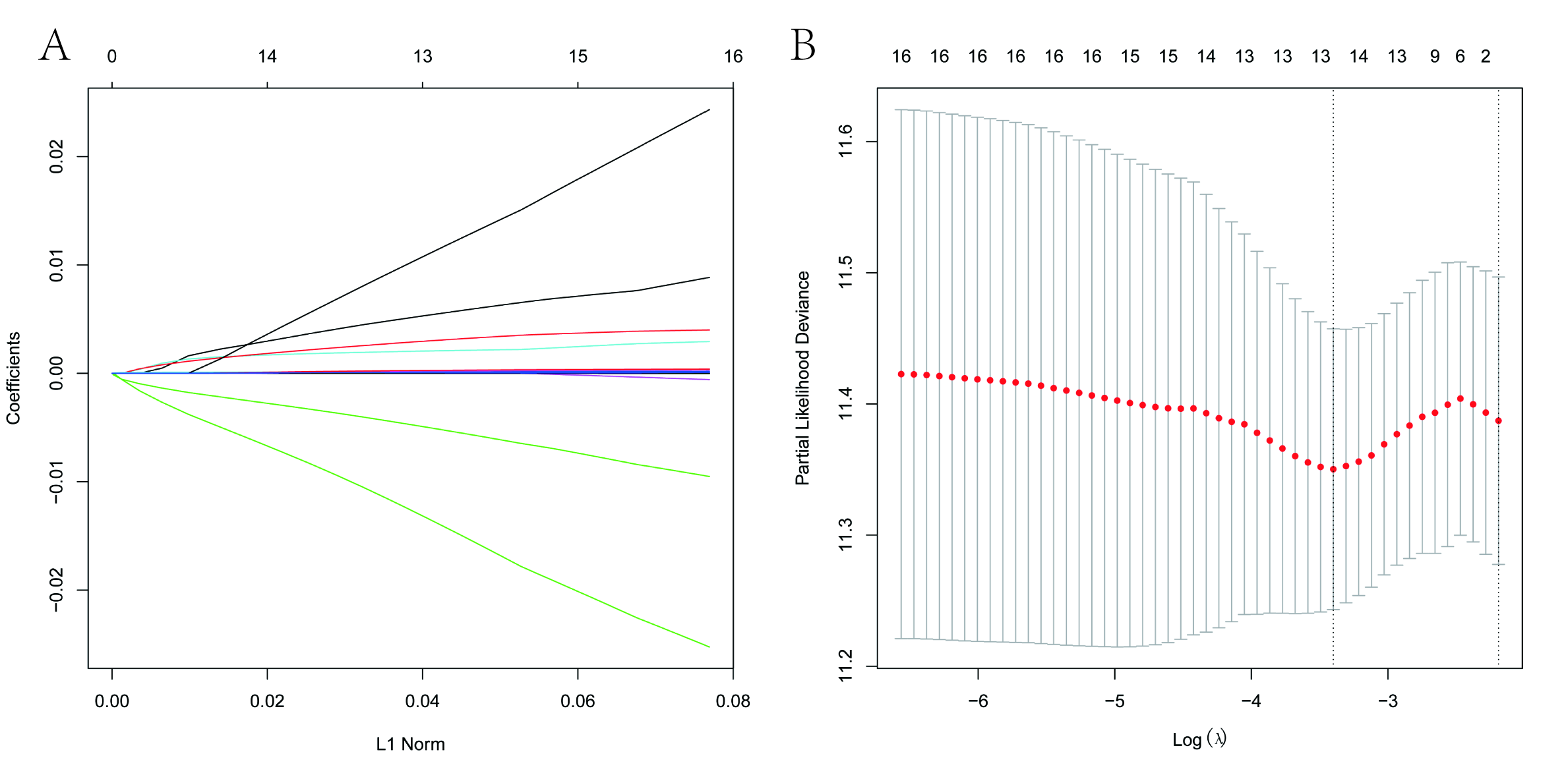

Supplement: Supplementary file 2 [file Image3.TIF]

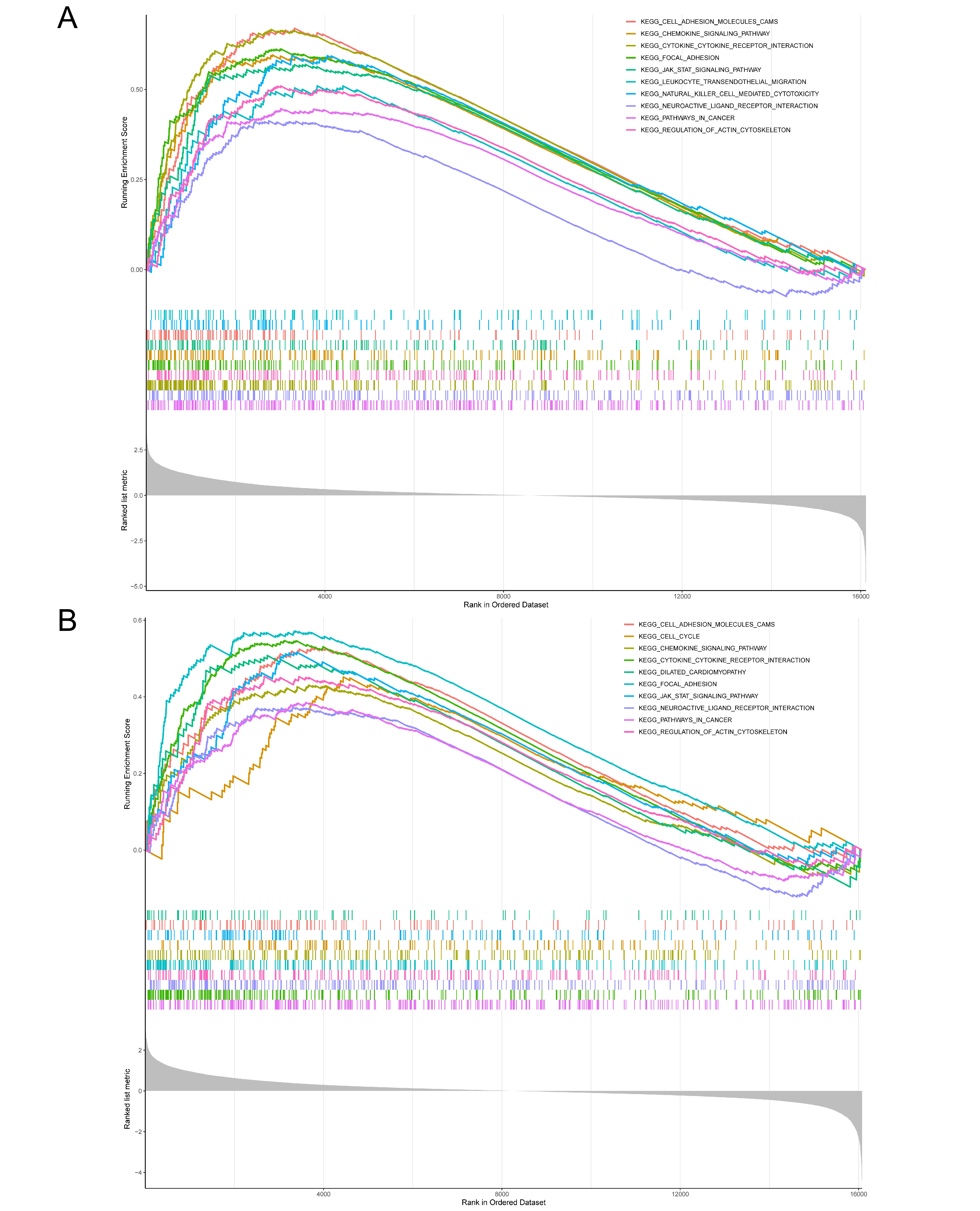

Supplement: Supplementary file 3 [file Image5.TIFF]

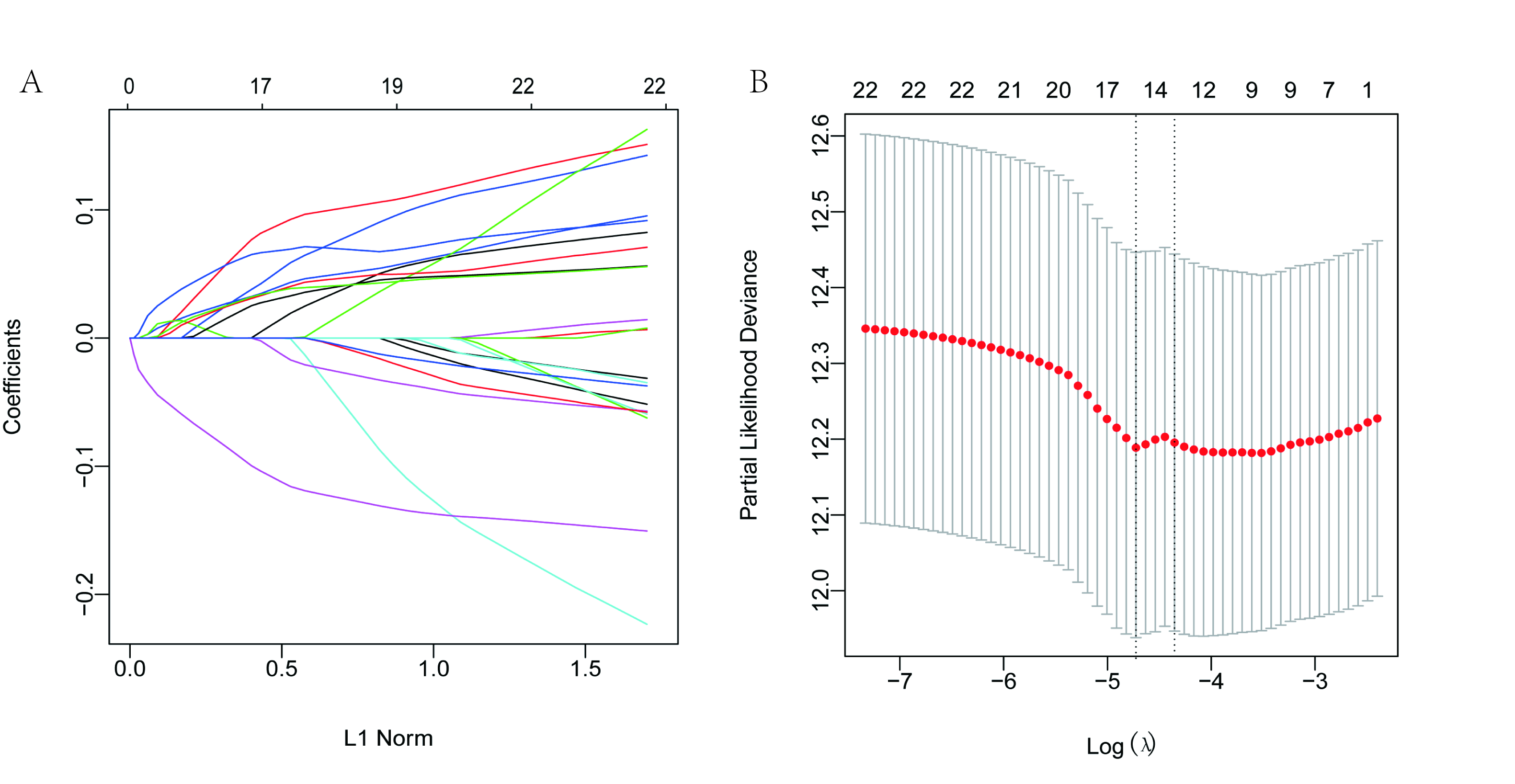

Supplement: Supplementary file 4 [file Image4.TIF]

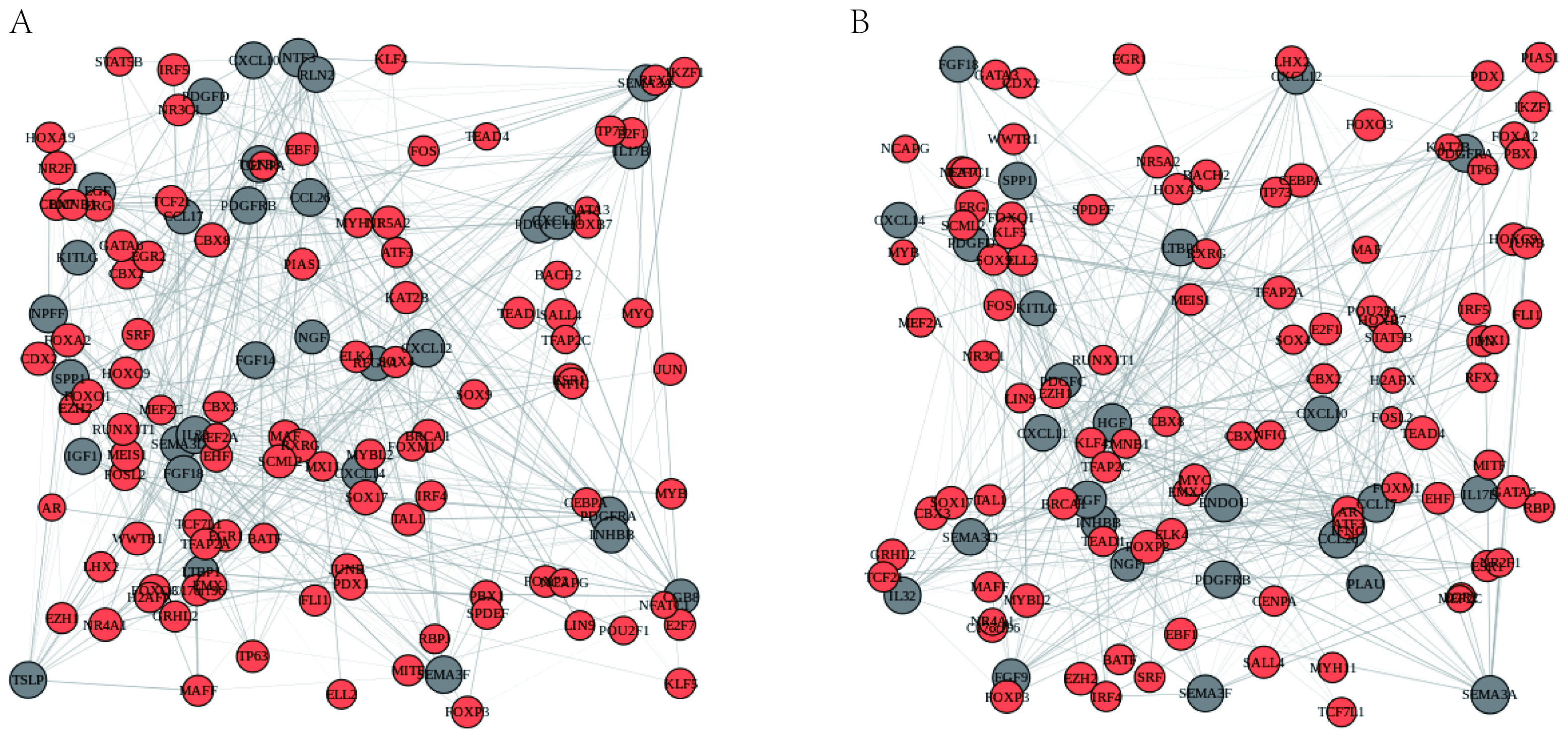

Supplement: Supplementary file 5 [file Image2.TIF]

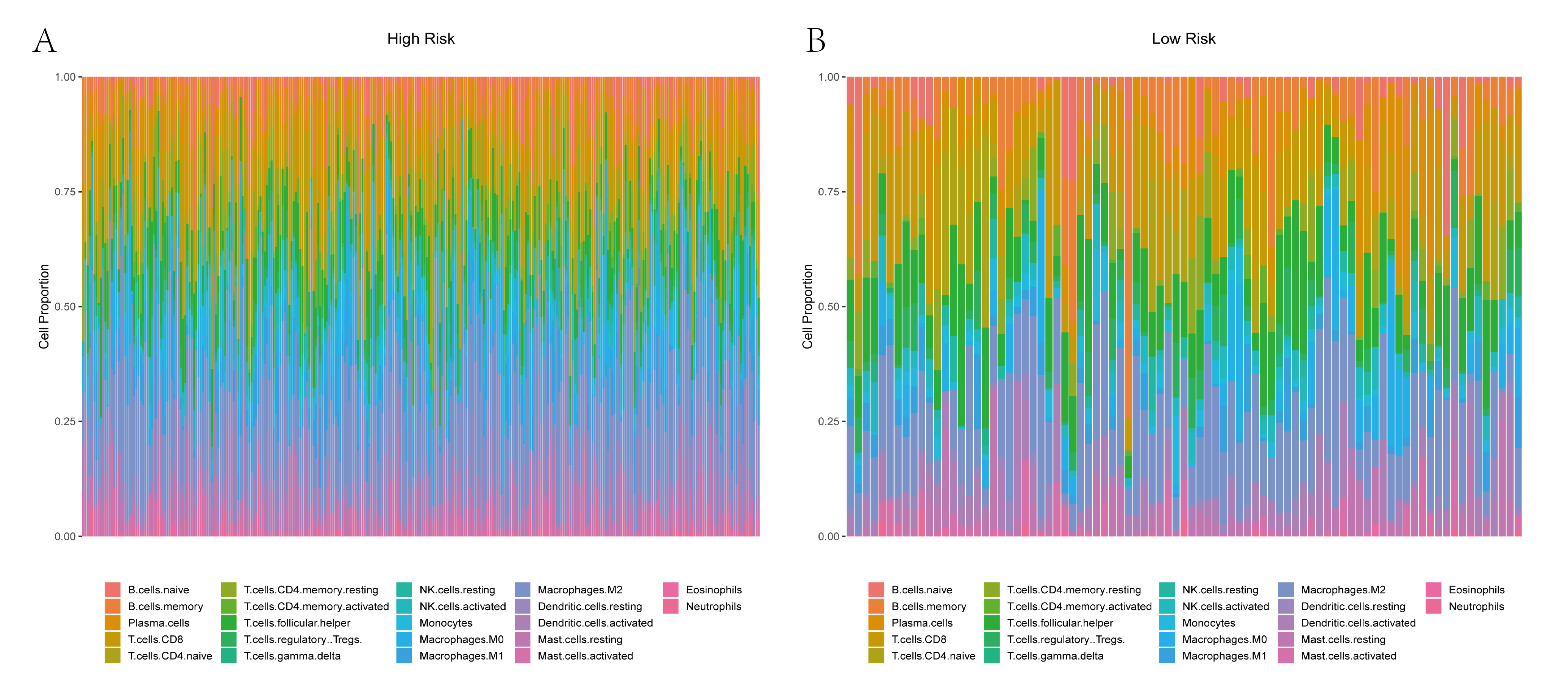

Supplement: Supplementary file 6 [file Image7.TIF]

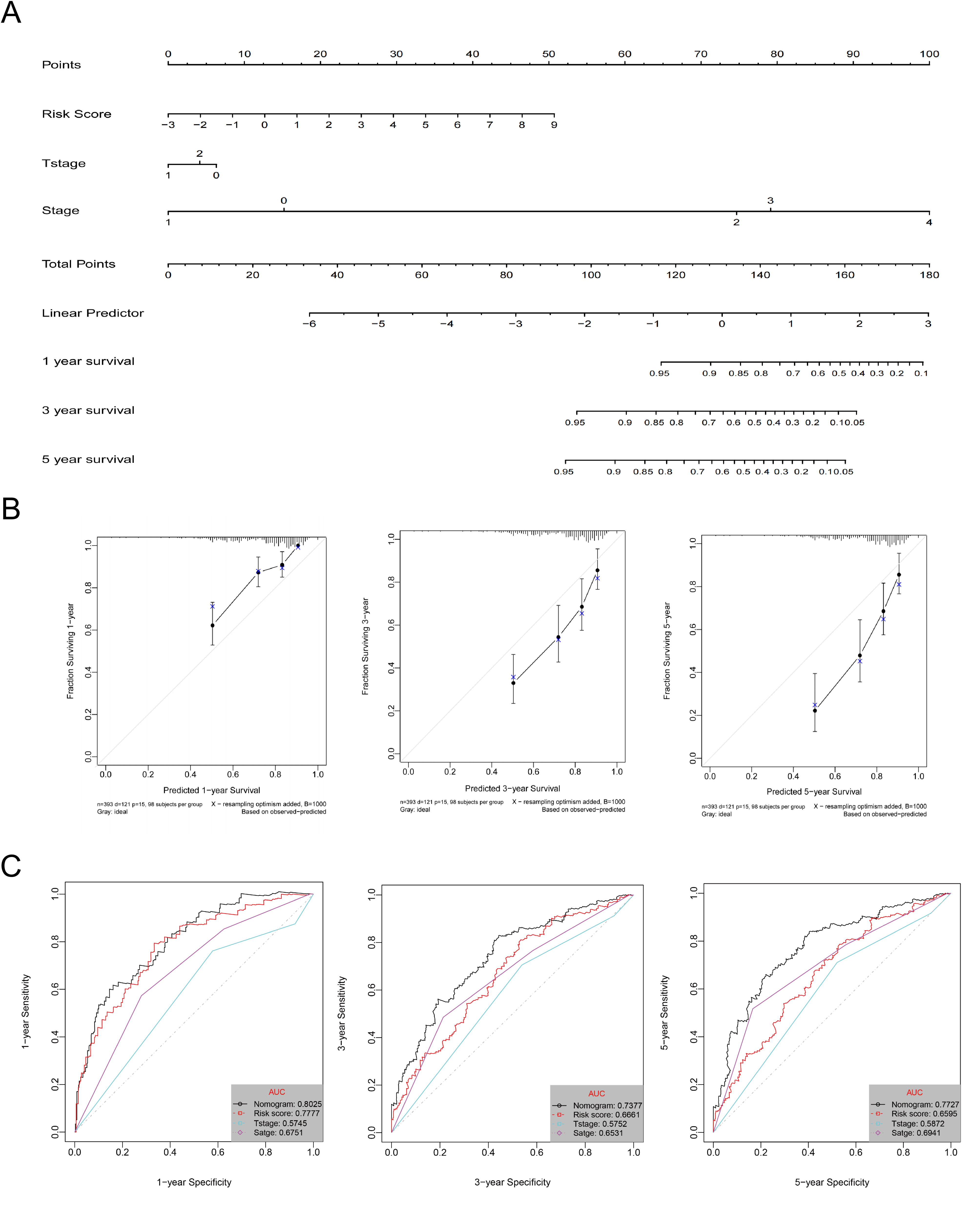

Supplement: Supplementary file 8 [file Image6.TIFF]

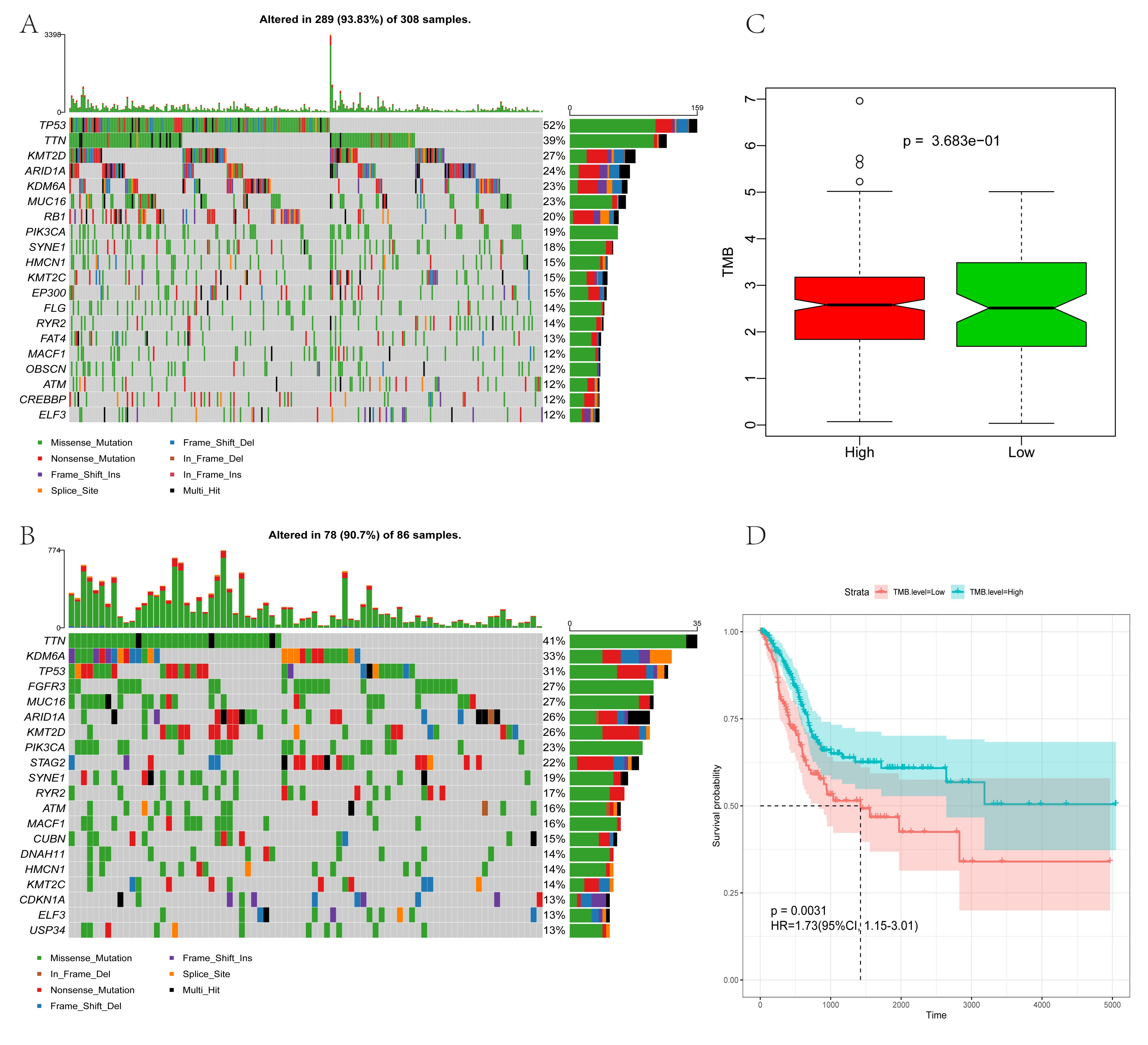

Supplement: Supplementary file 9 [file Image8.JPEG]
