# Supplementary material for: Immune Signature-Based Risk Stratification and Prediction of Immunotherapy Efficacy for Bladder Urothelial Carcinoma
Source: Front Mol Biosci. 2021 Dec 24;8:673918. doi: 10.3389/fmolb.2021.673918 (PMC8739239; doi:10.3389/fmolb.2021.673918)
Supplement: Supplementary file 7 [file DataSheet2.docx]

**Supplementary figures**

**Supplementary Figure S1**. Identification of DEGs between tumor and normal bladder tissues (1854 upregulated and 3145 downregulated genes). **(A)** Heatmap of DEGs. **(B)** Volcano plot of DEGs.

**Supplementary Figure S2 |** Interaction networks between DE-TFs and DE-IRGs. **(A)** Interaction networks between DE-TFs and 31 DE-IRGs related to OS in patients with BLCA. **(B)** Interaction networks between DE-TFs and 27 DE-IRGs related to DFS in patients with BLCA.

**Supplementary Figure S3 |** Construction of the 13-IRG signature. **(A)** LASSO coefficient profiles of 13 selected IRG signatures in 5-fold cross validation. The graph shows the change track of each independent variable; the horizontal axis represents the log value of the independent variable lambda, and the vertical axis represents the coefficient of the independent variable. **(B)** Partial likelihood deviance revealed by the LASSO regression model in the 5-fold cross validation. The model is optimal when λ = 0.03021.

**Supplementary Figure S4 |** Construction of the 15-IRG signature. **(A)** LASSO coefficient profiles of 15 selected IRG signatures in 5-fold cross validation. The graph shows the change track of each independent variable; the horizontal axis represents the log value of the independent variable λ, and the vertical axis represents the coefficient of the independent variable. **(B)** Partial likelihood deviance revealed by the LASSO regression model in the 5-fold cross validation. The model is optimal when λ = 0.01112.

**Supplementary Figure S5 |** KEGG enrichment analysis of DEGs between the high- and low-risk groups based on OS **(A)** and DFS data **(B)**. Top: broken line graph of gene enrichment score. Middle: the genes under the gene set are marked with lines. Bottom: rank value distribution of all genes.

**Supplementary Figure S6**. **(A)** Nomogram for predicting the risk of DFS. **(B)** Calibration plots of the nomogram for 1-, 3-, and 5-year DFS prediction. **(C)** Time-dependent ROC curves of DFS at 1, 3, and 5 years.

**Supplementary Figure S7**. Characterization of immune cell infiltrate in high- **(A)** and low-risk groups **(B)** based on the 15-IRG signature.

**Supplementary Figure S8**. Differences in TMB between high- and low-risk groups based on DFS. **(A)** Mutation profile of high-risk groups. **(B)** Mutation profile of low-risk groups. **(C)** Box plot of TMB score in high- and low-risk groups. **(D)** Survival curve of high- and low-TMB score group.

**Supplementary Table S1.** The complete clinical and survival information of 407 samples.

|  |  | TCGA-BLCA |
| --- | --- | --- |
| Survial |  |  |
| OS | Censored | 229 |
|  | Dead | 178 |
| DFS | Censored | 272 |
|  | Dead | 122 |
|  | unknown | 13 |
| Age | Age>60 | 300 |
|  | Age<=60 | 107 |
| Gender | Female | 106 |
|  | Male | 301 |
| M_stage | M0 | 196 |
|  | M1 | 11 |
|  | MX | 197 |
| N_stage | unknown | 3 |
|  | N0 | 236 |
|  | N1 | 46 |
|  | N2 | 75 |
|  | N3 | 8 |
|  | NX | 36 |
|  | unknown | 6 |
| T_stage | T0 | 1 |
|  | T1 | 3 |
|  | T2 | 109 |
|  | T3 | 193 |
|  | T4 | 58 |
|  | TX | 1 |
|  | unknown | 32 |
| stage | stage_I | 2 |
|  | stage_II | 130 |
|  | stage_III | 139 |
|  | stage_IV | 134 |
|  | stage_no | 2 |
| smoke | smoke_YES | 285 |
|  | smoke_NO | 109 |
|  | smoke_UN | 13 |

**Supplementary Table S2.** Univariate and multivariate Cox analysis in each group of BLCA patients with DFS.

| Variables | Univariate analysis | | | Multivariate analysis | | |
| --- | --- | --- | --- | --- | --- | --- |
|  | HR | 95% CI | P-value | HR | 95% CI | P-value |
| training group |  |  |  |  |  |  |
| Age (＜60 vs ≥60) | 1.018 | 0.997-1.041 | 0.097 | 1.014 | 0.992-1.037 | 0.223 |
| Gender (female vs male) | 0.759 | 0.475-1.214 | 0.259 | 0.701 | 0.435-1.129 | 0.144 |
| Tstage (T1/T2 vs T3/T4) | 1.308 | 0.897-1.906 | 0.151 | 0.631 | 0.420-0.950 | 0.027 |
| Nstage (N0 vs N1/N2/N3) | 1.565 | 1.267-1.935 | 0 | 1.17 | 0.813-1.684 | 0.398 |
| Stage (I/II vs III/IV) | 2.054 | 1.539-2.742 | 0 | 1.836 | 1.137-2.967 | 0.013 |
| Smoke_level (YES vs NO) | 1.064 | 0.718-1.577 | 0.754 | 1.038 | 0.705-1.530 | 0.849 |
| Riskscore (high/low) | 4.314 | 3.009-6.186 | 0 | 3.79 | 2.595-5.535 | 0 |
|  |  |  |  |  |  |  |
| entire group |  |  |  |  |  |  |
| Age (＜60 vs ≥60) | 1.019 | 1.001-1.037 | 0.037 | 1.016 | 0.998-1.035 | 0.082 |
| Gender (female vs male) | 0.885 | 0.596-1.317 | 0.551 | 0.939 | 0.617-1.427 | 0.767 |
| Tstage (T1/T2 vs T3/T4) | 1.426 | 1.040-1.957 | 0.022 | 0.751 | 0.537-1.050 | 0.094 |
| Nstage (N0 vs N1/N2/N3) | 1.543 | 1.296-1.838 | 0 | 0.953 | 0.710-1.279 | 0.747 |
| Stage (I/II vs III/IV) | 2.027 | 1.597-2.573 | 0 | 2.19 | 1.485-3.231 | 0 |
| Smoke_level (YES vs NO) | 1.322 | 0.917-1.904 | 0.122 | 1.226 | 0.852-1.766 | 0.272 |
| Riskscore (high/low) | 1.394 | 1.241-1.565 | 0 | 1.286 | 1.129-1.466 | 0 |
